# Music and sedation relieve intraoperative stress: A randomized controlled trial

**DOI:** 10.1371/journal.pone.0325038

**Published:** 2025-05-28

**Authors:** Kaoru Yamashita, Toshiro Kibe, Akari Uto, Shusei Yoshimine, Minako Uchino, Mitsutaka Sugimura

**Affiliations:** 1 Department of Dental Anesthesiology, Field of Oral and Maxillofacial Rehabilitation, Advanced Therapeutics Course, Graduate School of Medical and Dental Sciences, Kagoshima University, Kagoshima, Japan; 2 Department of Oral and Maxillofacial Surgery, Field of Oral and Maxillofacial Surgery, Advanced Therapeutics Course, Graduate School of Medical and Dental Sciences, Kagoshima University, Kagoshima, Japan; Universidade de Trás-os-Montes e Alto Douro: Universidade de Tras-os-Montes e Alto Douro, PORTUGAL

## Abstract

Music is useful for relieving anxiety; however, its impact on autonomic nervous system activity and circulatory dynamics during tooth extraction surgery under intravenous sedation is unclear. We conducted a prospective randomized controlled trial to compare the effects of music listening on circulatory dynamics and autonomic nervous system activity during the extraction of impacted mandibular third molars under intravenous sedation. We hypothesized that music listening combined with intravenous sedation would enhance stress reduction. In this randomized controlled trial, 34 women aged 20–40 years were assigned to a music or control group. Patients in the music group listened to music during tooth extraction under intravenous sedation. Autonomic nervous system activity, heart rate, and systolic blood pressure were assessed in both groups. Statistical significance was set at p < 0.05. Systolic blood pressure and autonomic nervous system activity were not significantly different between the two groups. However, the heart rate during the separation of the tooth crown, extraction, and suturing was significantly lower in the music group than in the control group (p < 0.05). Within-group systolic blood pressure comparisons showed significant differences during treatments between the groups. Intraoperative music listening may enhance the stress-reducing effects of intravenous sedation, as auditory intervention attenuates blood pressure variability and increments in heart rate even when the level of consciousness is reduced by sedation. The combination of intravenous sedation and music listening may be useful for reducing stress during dental treatment.

**Trial registration:**

UMIN Clinical Trials Registry, UMIN 000054970

## Introduction

Dental treatment can cause changes in autonomic nervous system (ANS) function and circulatory dynamics due to dental noise, pain, anxiety, and tension, leading to medical emergencies, such as abnormal hypertension and vasovagal reflexes [1,2]. Nonpharmacologic interventions, such as listening to music and verbal communication, as well as pharmacologic interventions with sedatives have been used to reduce these risks [3–5]. Listening to classical music during dental treatment may stabilize ANS and circulatory dynamics while relieving anxiety and tension [6].

In previous studies, classical music has been widely used to relax patients during dental treatment and has been shown to significantly reduce patient anxiety [7–9]. Furthermore, classical music is thought to influence physiological and psychological responses [10–15].

Furthermore, intravenous sedation (IVS) during dental treatment is thought to decrease the level of consciousness and produce an anxiolytic effect [16–18]. Tooth extractions, especially impacted mandibular third molar extractions, are stressful procedures for patients [19–21].

We previously reported that sedation or listening to music during extraction of an impacted mandibular third molar can decrease sympathetic nervous system (SNS) activity [22,23]. However, the specific effects of listening to music during sedation on ANS activity and circulatory dynamics remain unclear. Therefore, we hypothesized that listening to music, combined with IVS, would enhance stress reduction. In this study, we analyzed ANS activity and circulatory dynamics with the aim of investigating the usefulness of the combination of listening to music and IVS during tooth extraction.

## Materials and methods

### Ethics approval

**Fig 1:**
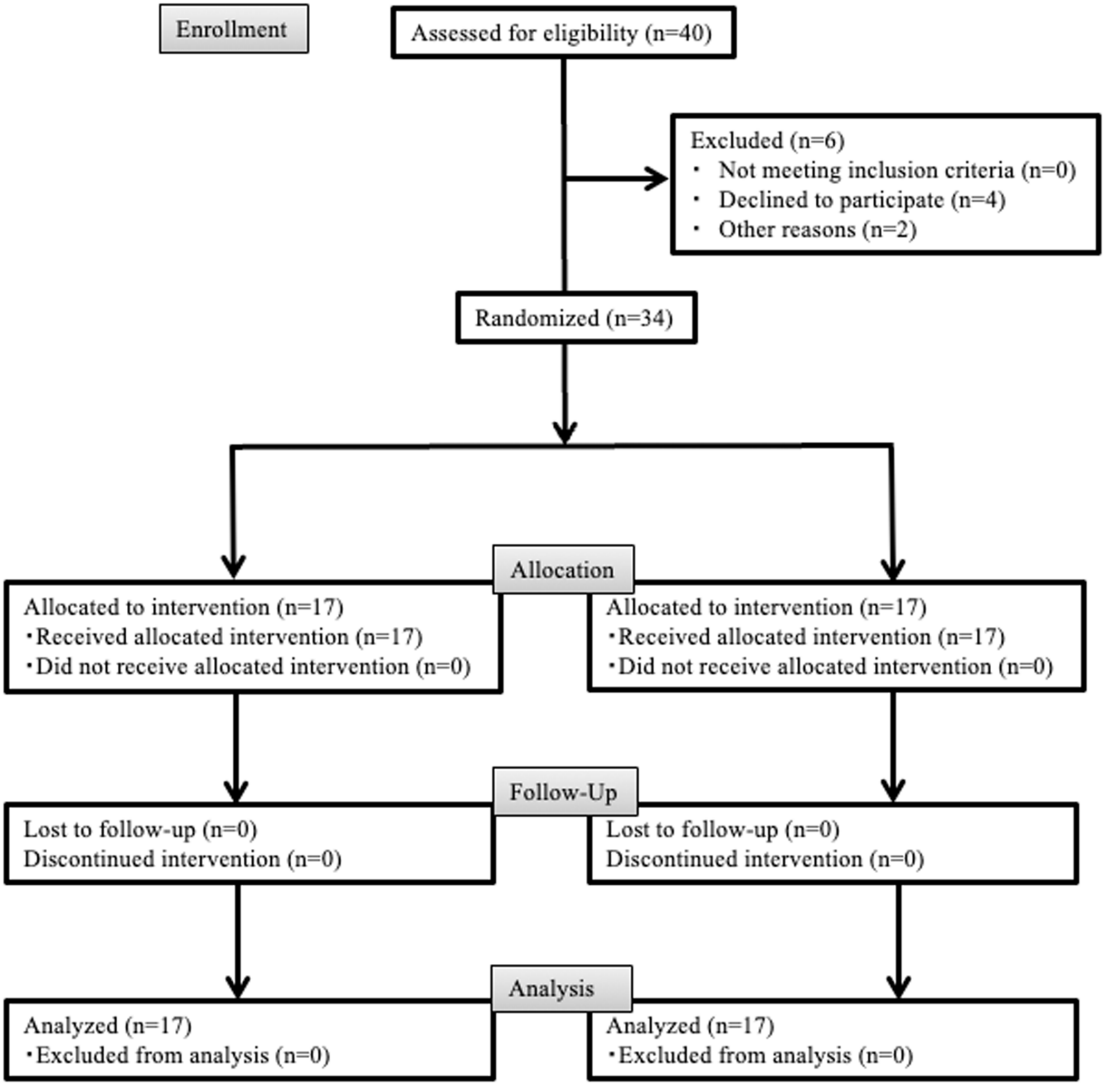
Consolidated Standards of Reporting Trials study design and CONSORT checklist. Local, local anesthetic injection; separation, tooth crown separation; extraction, extraction of the impacted mandibular third molar.

This study was approved by the Kagoshima University Hospital Clinical Research Ethics Review Committee (No. 180047) and adhered to the Consolidated Standards of Reporting Trials 2010 guidelines. Written informed consent was obtained from all patients. The study was conducted in accordance with the Declaration of Helsinki on Medical Protocols and Ethics. The study design (Fig 1) was a randomized controlled trial (prospective, randomized, open, blinded end-point study). Our study followed the model described by Yamashita et al. in 2019 [22]. The trial was registered with the UMIN Clinical Trials Registry (Clinical Trial Registration Number: UMIN 000054970).

### Study design and patient recruitment

Forty women aged 20–40 years scheduled for extraction of impacted mandibular third molars between December 2019 and November 2021 at Kagoshima University Hospital were enrolled in the study. The required sample size was calculated using G*Power software [24] by performing power analysis (α = 0.05, β = 0.2), using Mann–Whitney U test, and considering an effect size of 1. The effect size was calculated based on LF/HF values from our previous study [22].

As the required sample size was 34 participants, the number of participants was set at 40 to account for possible dropouts. Patients were randomly assigned to either a music or control group (n = 17 each) using the envelope technique. The department of biostatistics randomized and prepared the envelopes; the study authors had no access to the randomization codes. The examiners and surgeons were blinded to the patient group allocation. At the start of the session, a dentist not involved in the measurements fitted all patients with active noise-canceling headphones before the surgeon began treatment. The headphones’ active noise-canceling mode was turned on in the music group but not in the control group.

The exclusion criteria were diabetes, tobacco use, cardiovascular disease, diseases affecting the ANS, and prescription drug use [22,25–27]. Patients were instructed to avoid consumption of caffeine-containing beverages 24 h before the start of the procedure and to refrain from engaging in exercise and special diets for 6 h before the procedure [25–28].

### Study protocol

**Fig 2:**
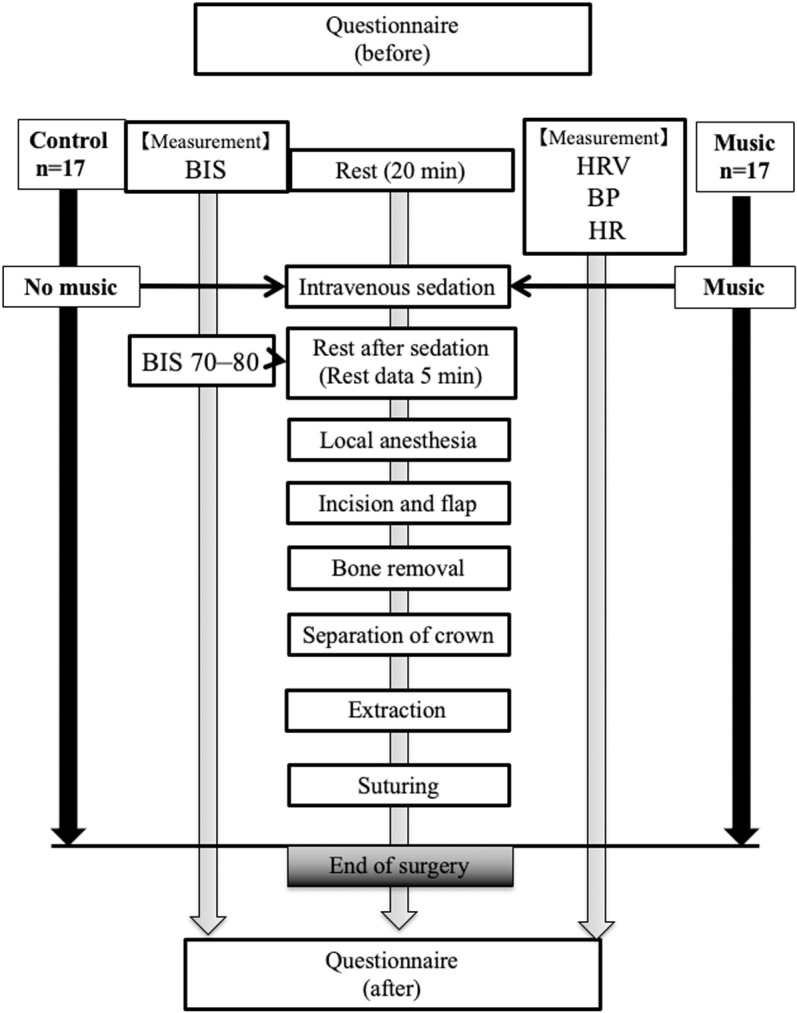
Study protocol. Local, local anesthetic injection; separation, tooth crown separation; extraction, extraction of the impacted mandibular third molar. BIS, bispectral index; HR, heart rate; HRV, heart rate variability; BP, blood pressure.

In the music group, patients listened to music during IVS; in the control group, they did not listen to music. Fig 2 shows the study protocol. Each patient sat in a semi-Fowler’s position on a dental chair in a noiseless clinic room maintained at 24 °C [25]. Heart rate (HR), HR variability (HRV), systolic blood pressure (SBP), and oxygen saturation were monitored during treatment. Pretreatment anxiety levels were assessed using the Modified Dental Anxiety Scale (MDAS) and the State-Trait Anxiety Inventory (STAI) [22]. The respiratory rate was measured silently at regular intervals during treatment.

A 22-G catheter was inserted into a vein in the left dorsal hand, and Ringer’s acetate solution was administered via infusion. Patients were instructed to rest with their eyes closed until the start of treatment. Midazolam (0.05 mg/kg) and propofol were then administered to achieve a bispectral index (BIS) of 70–80. Propofol doses were adjusted to maintain the BIS within this range during treatment. Before starting the treatment, the first 5 min of resting measurements, when the BIS fell to 70–80 under IVS, were used as control values [3,23,29]. All patients wore headphones throughout the perioperative period for data collection. Patients in the music group listened to a specific piece of music (Symphony No. 2 in E minor, Op. 27: 3rd movement Adagio, by Sergei Rachmaninoff) [22]. The local anesthetic used was 2% lidocaine with 1:80,000 epinephrine. All extractions were performed by an oral–maxillofacial surgical specialist to ensure standardized surgical conditions and procedures and manage patients’ stress levels [22].

### Sedation levels

Sedation levels were assessed using the BIS (70–80) and the Observer’s Assessment of Alertness/Sedation (scores 1–2) via a BIS monitor (Nihon Kohden, Tokyo, Japan) [16,18].

### ANS activity and circulation

To minimize the effects of circadian rhythms, we started conducting assessments at 14:00. HR and SBP were measured throughout the study; MemCalc-Makin2 software (GMS, Tokyo, Japan) was used for HRV analysis [9,21,29–35]. The power spectrum of heartbeat intervals was classified using frequency analysis into low (LF: 0.04–0.15 Hz) or high frequencies (HF: 0.15–0.4 Hz). LF is mediated by the SNS and parasympathetic nervous system, whereas HF is mediated only by the parasympathetic nervous system. Thus, the LF/HF ratio served as an SNS index and HF as a parasympathetic nervous system index [30]. SBP was assessed noninvasively every 2 min during the study.

### Questionnaire

Psychological testing was performed preoperatively using the MDAS and STAI. The STAI-State scale (STAI-S) assesses a transient situational response to “how I feel right now” for an anxiety-provoking event, whereas the STAI-Trait scale (STAI-T) assesses a relatively stable response tendency to an anxiety-provoking experience, such as “how I usually feel” in general. MDAS scores ≥19 are associated with “strong” dental phobia, whereas STAI-S scores ≥41 for men and ≥42 for women and STAI-T scores ≥44 for men and ≥45 for women are associated with “high” phobia.

### Statistical analyses

HRV and circulation data are expressed herein as ratios to resting measurements (control values) at specific time points (administration of local anesthesia, incision and flap reflection, bone removal, separation of tooth crown, and extraction). Data were analyzed using GraphPad Prism (version 6; GraphPad Software, La Jolla, CA, USA).

Changes in the control and music groups at each treatment (within-group) were tested using the Friedman test, with the Steel–Dwass test used for post-hoc tests, and comparisons between the control and music groups at each treatment were performed using the Mann–Whitney U test. The threshold for statistical significance was set at p < 0.05.

## Results

Four patients declined to participate and two were excluded because they subsequently received additional treatment (Fig 1). In total, 34 patients were randomly assigned to the control or music group (Fig 2). No significant differences were observed in age, height, weight, amount of local anesthetic used, duration of surgery, duration of anesthesia, or psychological test results between the groups (Table 1).

**Table 1:** Clinical characteristics of the patients.

	Control (n = 17)	Music (n = 17)	Total (n = 34)	95% CI	p-value
**Age (y)**	26.35 ± 3.16	25.53 ± 2.96	25.94 ± 3.04	24.92–26.96	0.38
**Height (cm)**	159.7 ± 4.61	160.02 ± 3.97	159.54 ± 4.35	158.43–161.28	0.74
**Weight (kg)**	53.35 ± 8.84	50.28 ± 4.5	51.82 ± 7.08	49.44–54.2	0.26
**Local anesthesia (mL)**	4.49 ± 0.79	4.26 ± 1.07	4.38 ± 0.93	4.06–4.69	0.38
**Operation time (min)**	44.65 ± 10.69	42.53 ± 13.61	43.59 ± 12.1	39.52–47.65	0.67
**MDAS scores**	12 ± 4.3	10.76 ± 4.79	11.38 ± 4.53	9.86–12.91	0.31
**STAI-S scores**	46.65 ± 7.83	42.82 ± 11.36	44.74 ± 9.8	41.44–48.03	0.12
**STAI-T scores**	54.18 ± 8.89	57.35 ± 9.92	55.76 ± 9.41	52.6–58.93	0.39

Data are presented as the mean±standard deviation. MDAS, STAI-S, and STAI-T scores are baseline values. MDAS, Modified Dental Anxiety Scale; STAI-S, State–Trait Anxiety Inventory-State; STAI-T, State–Trait Anxiety Inventory-Trait; CI, confidence interval. Statistical analysis: Mann–Whitney U test, p < 0.05

**Fig 3:**
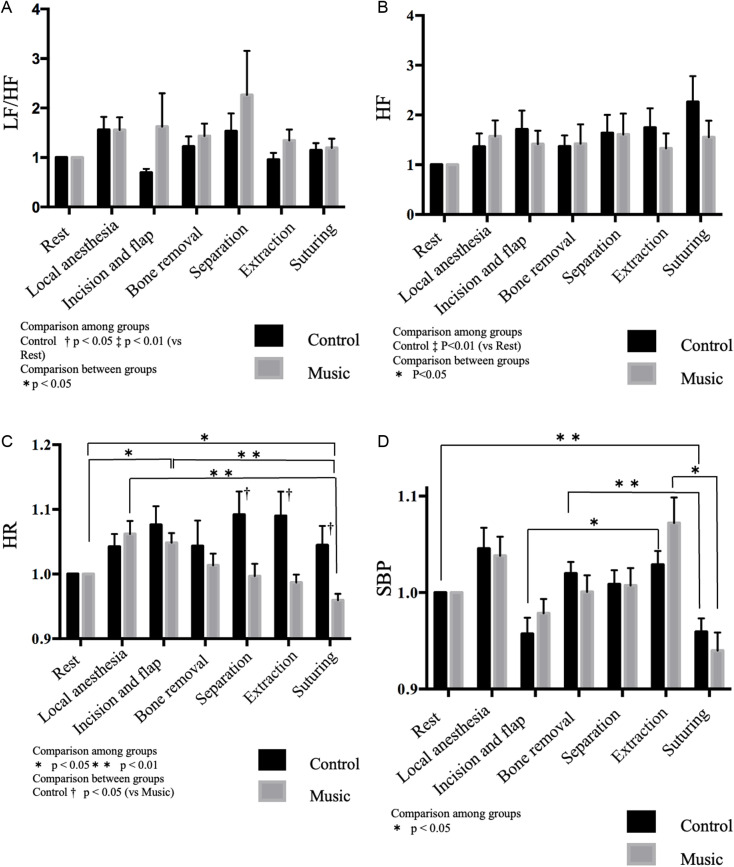
Comparisons between and within groups. (a) Comparisons of LF/HF ratios. (b) Comparisons of HF component during tooth extraction. (c) Comparisons of HR during tooth extraction. Comparison between groups: control (vs. music) † p < 0.05. Comparison within groups: control *p < 0.05, **p < 0.01. (d) Comparisons of SBP during tooth extraction. Comparison within groups: *p < 0.05, **p < 0.01. Friedman and Steel–Dwass tests were used for intra-group comparisons; Mann–Whitney U test used for inter-group comparisons. Data presented as mean and standard error of mean. LF, low frequency; HF, high frequency; HR, heart rate; HRV, heart rate variability; SBP, systolic blood pressure.

Additionally, no significant differences were observed in the LF/HF ratio or HF values during tooth extraction between the groups (Figs 3a, b). However, the HR was significantly lower in the music group than in the control group during separation of the tooth crown, extraction, and suturing (p < 0.05). No significant differences were observed in HR during surgery between the groups. During incision and flap reflection, the HR was significantly higher in the music group than in the control group (p < 0.05), but it was significantly lower in the music group during suture than at rest (p < 0.05). The HR during suturing was also significantly lower than during the administration of local anesthesia (p < 0.01; Fig 3c).

SBP did not significantly differ between groups (p < 0.05), although within-group comparisons showed significant differences between treatments. In the control group, the SBP during suturing was significantly lower than at rest and during bone removal (p < 0.01). Furthermore, SBP was significantly higher during extraction than during incision and flap reflection (p < 0.01). In the music group, SBP was significantly lower during suturing than during bone removal (p < 0.05; Fig 3d).

## Discussion

The main finding of this study is that auditory intervention attenuates blood pressure variability and increments in HR, even during sedation, indicating that IVS enhances the intraoperative stress reduction effects of intraoperative sedation. Tooth extraction compresses the periodontal ligament and has a stimulating effect on the body [36]. In the present study, music listening attenuated the variability in blood pressure and HR increases during tooth extraction and may contribute to reducing the onset of medical emergencies.

Lee et al. and Harikumar et al. reported this effect during colonoscopy [37,38]. In general, classical music is widely used to help patients relax during dental procedures [7,16], and it is thought to influence physiological and psychological responses [8,11–13,15,16]. In the present study, we used classical music composed by Rachmaninoff that has been shown to reduce SNS activity and anxiety during the extraction of impacted mandibular third molars [22]. Furthermore, a meta-analysis reported that listening to music reduces postoperative pain and anxiety, even under general anesthesia, indicating that brain regions responsible for hearing may still perceive music [39]. In the present study, we tested whether listening to music decreased SNS activity in sedated patients, as in our previous study, although these changes were not captured. A previous meta-analysis reported that the effects of music listening on pain and anxiety during general anesthesia are weaker than during consciousness [39]. Therefore, the effects of music may be more readily captured in the awake state. Sedatives also decrease HRV parameters [30,40–42], which may affect the ability to capture changes [17,43].

Although it is generally recognized that ANS activity and circulatory dynamics, such as HR, are related, under special circumstances, such as general anesthesia and sedation, anesthetics may disrupt the correlation between ANS and HRV by disrupting central nervous system integration processes and information transfer between the central nervous system and target organs [16]. Therefore, ANS variability was likely not detected in the present study owing to effects of the anesthetic. When interpreting HRV data, factors such as the ability of the brain’s neuromodulatory centers to receive and integrate information from peripheral receptors, the ability of sympathetic and vagal rhythms to reach the heart, and the responses of end organs to neuromodulation might explain the lack of correlation between HR and ANS activity [25]. Hence, the lack of correlation between HR and ANS activity in this study can be attributed to multiple factors as well as the effects of anesthetics. In the current study, HR was significantly lower during tooth crown separation, extraction, and suturing in the music group than in the control group. This aligns with findings of previous randomized clinical trials examining the effects of music during endodontic treatment, which reported significantly lower HR in music groups than in control groups [44].

For the determination of the necessary sample size, this study used a power of 0.8, a common value used in similar studies. Calculations were performed using effect sizes calculated from previous reports, and the sample size was set at 34. However, a smaller effect size for the same power may limit the generalizability of the results because of the larger sample size required. While a smaller effect size would require a larger sample size, as in a previous study [17], we set the effect size to 1 for this study based on a previous study conducted at our institution [23].

The main limitation of this study is the slight difficulty in interpreting the data because sensitivity to music is suppressed under sedation. However, we believe that the findings of this study, considering previous intervention studies using IVS or music alone, are valid given the reproducibility of the results [23,24]. Future research should include not only HRV data but also use other devices that measure ANS activity as well as other indicators to make a comprehensive determination. In the present study, the target patients were limited to women 20–40 years of age; however, ANS activity differs between men and women and, moreover, can vary between age groups. Therefore, to eliminate as many confounding factors as possible, this study was limited to patients of one sex and age range. Although only one piece of music was used to ensure consistency between conditions, music with faster tempos has been shown to increase SNS activity [45]; thus, the results obtained here may not be generalizable to studies using other genres of music or patient-preferred music.

## Conclusion

In summary, listening to music attenuated blood pressure fluctuations and HR changes even when the patient’s level of consciousness was reduced by sedation, indicating that IVS may be effective in reducing intraoperative stress. Furthermore, the combination of IVS and music listening may be useful in reducing stress during dental treatment. Further studies are needed to investigate these effects with other types of music or with patient-preferred music. The findings of this study may inform other surgeries requiring sedation, and further research is warranted.

## Supporting information

S1 Checklist CONSORT_checklist. (PDF)

S1 Protocol Clinical Research Protocol 1 ver. (DOCX)
