# Supplementary material for: Music and sedation relieve intraoperative stress: A randomized controlled trial
Source: PLoS One. 2025 May 28;20(5):e0325038. doi: 10.1371/journal.pone.0325038 (PMC12118882; doi:10.1371/journal.pone.0325038)
Supplement: S1 Protocol — (DOCX) [file pone.0325038.s002.docx]

Clinical Research Protocol ver. 1.0

Created on April 20, 2018

Medical Science Research Implementation Plan

〔Subject for study〕

Effects of listening to music during dental treatment under intravenous sedation on autonomic nervous system, electroencephalogram, and psychological state.

1. Background of the study and basis for scientific rationality

Intravenous sedation and listening to music during dental treatment are widely accepted as methods for providing safe dental treatment. However, it is unclear how listening to music during dental treatment under intravenous sedation affects the human body and psychology.

The purpose of this study is to determine how listening to music during dental treatment under intravenous sedation affects electroencephalogram, autonomic nervous function, and psychological state. Previous research reports have revealed that it is linked to parameters of the autonomic nervous system, electroencephalogram, and circulatory dynamics as an indicator of relaxation, and it is a common evaluation item. Therefore, it can be said that this study has scientific rationality.

2. Purpose and Significance of the Study

The purpose of this study is to determine the effects of listening to music under intravenous sedation on electroencephalograms, autonomic nervous function, and psychological aspects. The subjects were 40 patients who visited Our University Hospital, who were judged by dentists to require intravenous sedation tooth extraction at the Our University Hospital, and who expressed their intention to participate in this study. The primary evaluation items were electroencephalograms, autonomic nervous function, and psychological aspects. If the results obtained can be feedback to the dental clinical field, it is believed that even patients who are anxious about dental treatment will be able to receive comfortable dental treatment. Systemic complications caused by mental stress, such as vasovagal reflex and hyperventilation attacks, which are likely to occur during dental treatment, can be prevented. Furthermore, it is believed that systemic complications such as increased blood pressure, tachycardia attacks, arrhythmia, and angina attacks during dental treatment can be prevented in patients with cardiovascular diseases such as hypertension and ischemic heart disease. Therefore, the significance of this study is also considered to be great in terms of safe dental care.

3. Selection policy for research subjects (collaborators)

Patients undergoing tooth extraction under intravenous sedation at the Our University Hospital General Management Dental Treatment Department

3.1. Eligibility criteria

① Diagnosis (including diagnostic criteria): Cases in which the dentist judges that tooth extraction under intravenous sedation is necessary

② Disease stage: Cases in which the dentist judges that acute symptoms have subsided

③ Age, gender: 20-40 yers, women

④ Outpatients

⑤ Target period (time of diagnosis, hospitalization, and outpatient visit): Time of diagnosis and time of treatment

3.2. Exclusion criteria

(Exclusion criteria for influencing efficacy evaluation)

① Cases with diseases that affect the autonomic nervous system or electroencephalogram

② Cases with a history of smoking

③ Cases taking medication regularly

4. Research Methodology

4.1 Details of Research Methods

1) Specify the Sample/Information to Be Used for the Research

1. Specific Details

Forty patients will be randomly assigned to two groups of 20 patients each: control and music　 using the envelope method. Patients in the music group listened to music during tooth extraction.

Patients whose dentist determines that a tooth needs to be extracted will be included in the study.

The Modified Dental Anxiety Scale (MDAS) will be administered before the start of the treatment.

The State Trait Anxiety Inventory-Trait Anxiety Scale (STAI) psychological test will be administered before and at the end of the procedure. Recording of heart rate variability and circulatory dynamic will begin 15 minutes before the start of the procedure while wearing headphones, and will be completed at the end of the procedure.

After optimal sedation concentration under intravenous sedation, the procedure is performed by the same surgeon, local anesthesia is administered, and tooth extraction is initiated.

Measurement analysis during tooth extraction with music listening through headphones will also be performed, and the two groups, the control group and the music group, will be statistically compared and analyzed using the analysis software GraphPad Prism6.

2. Period of Time to Be Collected (Be Specific)

Date of approval - March 31, 2023

2) Analysis Method

Statistical comparative analysis using analysis software GraphPad Prism6.

3)

1. Test Drug/Propofol

Test machine/ EEG and ECG real-time analysis system MemCalc/Makin2

Test method/ music during dental treatment under intravenous sedation.

2. Dosage/Treatment Intensity, etc. //The volume of the music should be at a level that does not cause distress to the patient. //If there is a marked change in vital signs as a criterion for changing the treatment intensity, etc., discontinue the treatment.

For intravenous sedation, follow the guidelines for intravenous sedation of The Japanese Dental Society of Anesthesiology.

The patient should be given intravenous sedation in accordance with the guidelines of The Japanese Dental Society of Anesthesiology.

3.Criteria for Deferral, Dose Reduction, Withdrawal, and Suspension

Discontinue if the subject complains or if there is a marked change in vital signs.

The patient should be given intravenous sedation in accordance with the guidelines of The Japanese Dental Society of Anesthesiology.

4. Discontinuation and Completion Criteria

Criteria for Adverse Events to Be Stopped, Deterioration of the Underlying Disease, etc., and Criteria for Completion of the Study /

Discontinue if subject complains or if a prominent change in vital signs is observed.

5. Acceptable Concomitant Therapy

None.

6. Unacceptable Concomitant Therapy

None

7. Treatment Restrictions after Completion of the Intervention

None

4.2. Test schedule

All measurements will be taken on the day of treatment. A questionnaire will be conducted before and after the start of treatment. Parameters for autonomic nerves, electroencephalograms, and hemodynamics will be collected during treatment. Since no data will be collected before the test date, the number of days allowed for the test date to differ will not be determined.

4.3. Study period

Total study period: Date of approval - March 31, 2023

Registration period: Date of approval - March 31, 2022

Follow-up period: Case registration - 1 year

4.4. Target number of subjects and the basis for setting it

Number of cases: 40 cases who have given consent to this study

Bases for setting: α value 0.05, β value 0.2, effect size 1SD were used as the basis for setting the number of cases. It was set using Gpower.

5. Expected effects, side effects, or disadvantages to the study subjects, and responses to them and comprehensive evaluation of these

5.1. Effects

Participation in this study will not have a direct effect on the study subjects, but the knowledge gained from this study can contribute to the creation of a system that provides a better dental treatment environment for dental treatment patients.

5.2. Side effects or burden on the study subjects, as well as expected risks and how to deal with them, etc.

Listening to music before entering a sedative state cannot be said to be unpleasant depending on the genre or volume of the music, so if a patient complains, music listening will be immediately discontinued.

5.3. Response in the event of a serious adverse event

If a serious adverse event occurs, it will be dealt with promptly. If symptoms are judged to be unknown side effects or severe, they will be reported immediately to the principal investigator and the director of Our University Hospital in accordance with Our University Hospital's "Procedure for Serious Adverse Events."

5.4. Compensation for health damage caused by the research:

- If health damage occurs, it will be handled through medical insurance. No monetary compensation will be provided.

6. Response regarding the provision of medical care to research subjects after the research is completed

Since it cannot be said that there will be no physical disadvantages due to changes in physical condition or fatigue during the examination, if such a thing occurs, the examination will be immediately stopped and all possible measures will be taken, such as having the doctor or nursing staff treat the patient.

7. Other treatment methods

Treatment will be performed without allowing the patient to listen to music.

8. Possibility of obtaining important knowledge regarding the health of the research subjects and how to handle it (including incidental findings)As a result of the implementation of this study, there is a possibility that knowledge regarding the health of the research subjects will be obtained, but since this knowledge is still in the research process and it is unclear at this time to what extent it will affect the subjects' health, it will not be explained to the research subjects.

9. About the samples and information obtained

9.1. Method of storing samples and information

Medical information will be stored at the Our University Hospital for at least five years from the date on which the completion of the study is reported or three years from the date on which the final publication of the results of the study is reported, whichever is later. After the retention period has expired, the samples will be crushed into a form that cannot identify individuals and disposed of. Materials containing personal information will be stored in a locked storage facility and will be stored even after the study has ended, and will be used for new research approved by the ethical committee, only if the subject's consent has been obtained.

9.2. Method of Disposal of Samples and Information

After the retention period has expired, the samples will be crushed into a form that cannot identify individuals and disposed of.

9.3. Possible and expected content for future research that is not identified at the time of obtaining consent from the research subjects, etc.

Dental treatment is characterized by frequent iatrogenic noise such as turbines, engines, and vacuum sounds. Therefore, it is thought that the auditory stimulation of patients during dental treatment is highly related to autonomic nervous fluctuations. Therefore, in the future, there is a possibility that research will be conducted on the effects of the combined use of nitrous oxide and music on auditory electroencephalograms, psychological aspects, and various parameters related to the autonomic nervous system. If the samples and information are to be used for research other than the research being applied for this time, approval will be obtained separately from the relevant research ethics committee.

10. Source of funding, relationships with related organizations, and conflicts of interest

10.1. Source of funding

Expenses related to this study, such as costs related to data analysis, will be covered by research funds (educational and research infrastructure expenses) from Our University Hospital.

Other costs related to medical treatment and examinations will be borne by the patient within the scope of insured medical care.

10.2. Relationships with related organizations and conflicts of interest

This study will be conducted using the research funds mentioned in 10.1 above. As no funding or convenience has been provided by companies or other organizations for this study, there are no conflicts of interest to disclose in this study.

11. Financial burden and research cooperation fee incurred by participating in the study

No research cooperation fee will be paid

12. Monitoring and audit implementation system and implementation procedure

12.1. Monitoring

Implementation procedure: Monitoring will be conducted at the start of the experiment, three months after the start of the experiment, and the end of the experiment to check whether there are any problems with the volume from the start to the end of the study.

12.2. Audit

Implementation procedure: Audits will be conducted at the start of the experiment, three months after the start of the experiment, and the end of the experiment to check whether there are any problems with the volume from the start to the end of the study.

13. Procedures for obtaining informed consent, etc.

13.1. About informed consent

As per the attached explanatory document, the research subjects will be fully explained and, once they fully understand, will be asked to sign a consent form for participation in the research. In addition, it will be fully explained that the subject can withdraw consent at any time without suffering any disadvantage.

14. About handling of personal information

In order to completely protect the privacy of the research subjects, the principal investigator and research co-investigators will not publish any information that could identify individuals when announcing or publishing the results of the analysis. All materials obtained will be made into a correspondence table and anonymized, and will not be used for research other than that described in the research plan. If the materials are to be used for other research, consent will be obtained again.

15. Content of the relevant work and supervision method of the contractor when part of the research-related work is entrusted to another party

None

16. Response to inquiries from research subjects

The principal investigator will respond in good faith.

17. Contents and methods of reporting to the head of the research institution

① Changes in the implementation plan/As needed

② Research progress report/Every year around November

③ Research completion report/At the end of the research

④ Other matters will be reported without delay in compliance with the "Ethical guidelines for medical research involving human subjects."

17. Method of Disclosure of Research-Related Information

An overview of this study and its progress will be made public on UMIN-CTR (www.umin.ac.jp/ctr/).

In addition, when the results of the research are made public, they will be made public in a manner that prevents the identification of research subjects (collaborators).
